# A Post-Quantum Authentication and Key Agreement Protocol Based on Lattice-Based KEM for Secure Network Environments

**DOI:** 10.3390/e28050490

**Published:** 2026-04-24

**Authors:** Xiaoping Chen, Wangyu Wu, Guangmin Liang, Haonan Tan, Yicheng Yu

**Affiliations:** 1 School of Electronic and Communication Engineering, Shenzhen Polytechnic University, Shenzhen 518055, China; 2 School of Computer Science, University of Liverpool, Liverpool L69 3DR, UK

**Keywords:** post-quantum, authentication and key agreement protocol, Kyber, network security

## Abstract

In emerging environments such as cloud computing and the Internet of Things (IoT), secure authentication and key negotiation play a crucial role in protecting data transmitted over public networks. However, many existing authentication protocols are still designed based on classical public-key cryptography primitives, and quantum computing may threaten their security. To address this challenge, we propose a post-quantum authentication and key agreement protocol that uses the lattice-based Kyber key encapsulation mechanism (KEM). Our proposed protocol integrates cryptographic authentication, smart card protection, and post-quantum key encapsulation mechanisms, enabling mutual authentication between users and servers and securely establishing session keys. The security of the protocol is formally analyzed in the Real-or-Random (ROR) model under the random oracle assumption and the IND-CCA security of the underlying KEM scheme. Furthermore, through informal security analysis, we have further demonstrated that the protocol possesses important security properties, including anonymity, untraceability, perfect forward confidentiality, and resistance to known attacks. In addition, the computational cost and communication overhead of the proposed scheme are evaluated and compared with several representative authentication protocols. The results show that the proposed protocol can provide strong security while maintaining low computational cost and communication overhead.

## 1. Introduction

With the rapid development of cloud computing and Internet of Things (IoT) technologies, numerous devices and users now connect to remote servers over public networks. In these settings, secure remote user authentication and session key establishment are essential for protecting sensitive data and preventing unauthorized access. Therefore, authentication protocols are widely deployed to verify the legitimacy of communicating entities and to establish secure session keys for secure subsequent communications.

Traditional authentication and key agreement protocols usually use public-key cryptography to make communication secure over unsafe channels. However, the advent of quantum computing introduces fresh challenges to the sustained security of numerous traditional cryptographic methods. In recent years, post-quantum cryptography has drawn much attention. As a result, many researchers now work on building authentication protocols that stay secure against quantum adversaries.

As one of the main options for post-quantum security, lattice-based cryptography offers strong security and can be implemented efficiently. The National Institute of Standards and Technology (NIST) has standardized the CRYSTALS-Kyber key encapsulation mechanism as the ML-KEM algorithm. Its security depends on the difficulty of the Module-LWE problem. This gives a practical base for building post-quantum secure authentication and key agreement protocols. At the same time, modern secure network environments are increasingly characterized by dynamic and intelligent attack defense interactions. Recent studies have shown that, beyond cryptographic design itself, defense mechanisms such as moving target defense and game theoretic strategy making are becoming important in complex network security scenarios [1,2]. However, these higher-layer defense mechanisms still require a reliable authentication and session key establishment basis. This motivates the study of a practical post-quantum authentication protocol that can be deployed in conventional public network environments with low structural complexity.

**Figure 1:**
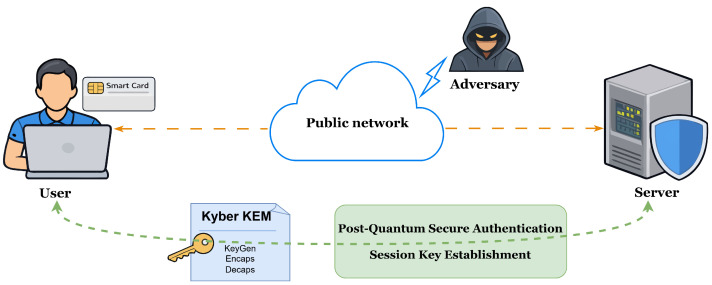
System model.

Based on these observations, we propose a new authentication and key agreement protocol that uses lattice-based KEM primitives for post-quantum security. In this work, we focus on this basic two-party setting in order to study the post-quantum authentication and session key establishment problem in a structurally simple and analytically tractable form before considering more complex multi-server or federated environments. Figure 1 illustrates the overall system architecture of the proposed post-quantum authentication protocol. The system involves two main entities: a user and a remote server communicating through a public network. The user utilizes a smart card together with a password to perform authentication, while the server employs the Kyber lattice-based key encapsulation mechanism (KEM) to establish a shared session key. Through the integration of password authentication, smart card protection, and post-quantum cryptographic primitives, the protocol enables secure mutual authentication and session key establishment in insecure network environments.

The main contributions of this work are summarized as follows:

- We propose a two-party post-quantum authentication and key agreement protocol that integrates password verification, smart-card-protected credentials, and the Kyber KEM into a unified framework. Unlike several existing post-quantum authentication schemes, the proposed design does not rely on a registration center, fuzzy extractors, or hybrid construction of classical and post-quantum cryptography, thereby reducing structural complexity.
- We design a dual KEM-based session key establishment mechanism in which the session key is jointly derived from a server-side encapsulated secret and an ephemeral encapsulated secret of the user. This design enables mutual authentication and strengthens session key freshness while preserving user identity protection over public channels.
- We prove the semantic security of the established session key in the Real-or-Random model under the random oracle assumption and the IND-CCA security of the underlying KEM by a sequence of games reduction tailored to the two encapsulated secrets used in the protocol.
- We evaluate the proposed scheme against representative related protocols and show that, by avoiding processing related to the fuzz extractor and other auxiliary components, the proposed protocol achieves lower computational cost while maintaining acceptable communication overhead and a broad set of security properties.

The remainder of this work is organized as follows: Section 2 provides an overview of previous research on authentication and key agreement protocols. Section 3 outlines the foundational cryptographic concepts and defines the adversary model. Section 4 offers a comprehensive explanation of the proposed protocol. Section 5 presents both formal and informal analyses of its security. In Section 6, we assess the performance of our scheme and compare it with existing protocols. Section 7 examines the implications and possible limitations of our approach. Lastly, Section 8 concludes the paper.

## 2. Related Works

Remote authentication protocols have been extensively studied for cloud computing, multi-server architectures, and Internet of Things (IoT) environments. Early authentication schemes mainly relied on passwords, smart cards, biometrics, and elliptic curve cryptography (ECC) to achieve lightweight authentication and session key establishment. For instance, Kumari et al. [3] proposed a biometric authentication scheme that is provably secure for multi-cloud environments. Kandar et al. [4] introduced a smart-card-based biometric authentication technique for multi-server environments. Mahmood et al. [5] further crafted a lightweight PUF-enabled authentication protocol for multi-server communication systems. These approaches improve usability and reduce authentication overhead, but they remain dependent on classical cryptographic assumptions.

To enhance privacy protection and device-level security, several works integrate biometric protection, biohashing, or physical unclonable functions (PUFs) into authentication protocols. Biohashing-based mechanisms aim to protect biometric templates during authentication, whereas PUF-based approaches bind credentials to hardware devices to mitigate cloning attacks. Recent studies also explore lightweight privacy-preserving authentication for IoT devices using hardware-assisted mechanisms [6,7]. Despite these improvements, most existing schemes [8] still rely on ECC-based cryptography, which may become vulnerable to quantum adversaries.

With the rapid development of post-quantum cryptography, lattice-based cryptographic primitives have attracted increasing attention. The CRYSTALS-Kyber key encapsulation mechanism proposed by Bos et al. [9] has become one of the most prominent lattice-based constructions and was standardized by NIST as ML-KEM in FIPS 203 [10]. These developments have motivated researchers to design post-quantum authentication protocols for various network environments.

Several recent studies adopt lattice-based primitives to construct quantum-resistant authentication protocols. Zhao et al. [11] proposed a password-authenticated scheme based on key consensus for IoT environments. Chen et al. [12] introduced a quantum-safe multi-server password-authenticated protocol providing user anonymity. Mrityunjay et al. [13] designed a post-quantum authentication protocol for wireless sensor networks. Pursharthi and Mishra [14] further proposed a post-quantum framework for secure communication in multi-server networking.

In application-oriented scenarios, several studies have investigated post-quantum authentication for emerging systems. Mansoor et al. [15] proposed PQCAIE, a post-quantum authentication scheme for IoT-based e-health systems. Franco et al. [16] explored a cloud-based multifactor authentication architecture using post-quantum cryptography and trusted execution environments. Bianchi et al. [17] introduced DynamiQS, a quantum-secure authentication protocol for dynamic charging systems in vehicular networks.

Hybrid authentication frameworks have also been explored to support the migration toward post-quantum security. Braeken et al. [18] proposed a flexible hybrid multi-factor authentication and key agreement framework combining ECC and post-quantum KEM primitives. In addition, Sikeridis et al. [19] and Schwabe et al. [20] analyzed the practical deployment of post-quantum cryptography in secure communication protocols such as TLS, highlighting the feasibility of integrating lattice-based primitives into real-world systems.

Shamshad et al. [21] developed a PUF-assisted authentication protocol for multi-server environments using hardware-based primitives to enhance device-level security. Meanwhile, Wen et al. [22] proposed a post-quantum secure multi-factor authentication protocol for multi-server architectures that integrates a fuzzy extractor with the Kyber key encapsulation mechanism. These studies demonstrate the growing interest in combining post-quantum cryptography with lightweight authentication mechanisms. Although these schemes provide important improvements, many of them rely on additional architectural components such as fuzzy extractors, registration centers, or hybrid cryptographic mechanisms, which may increase system complexity.

In summary, the related literature shows a clear development trend from classical authentication schemes based on passwords, smart cards, biometrics, and ECC to more recent post-quantum authentication designs based on lattice cryptography and KEM primitives. Quantum cryptography is another important direction for resisting quantum threats, and related studies have also demonstrated its potential in secure communications [23]. However, such approaches usually rely on dedicated quantum communication infrastructure and differ from the deployment setting of conventional public network authentication protocols. Although these studies have significantly improved resistance against quantum attacks, many existing post-quantum schemes still rely on additional components such as fuzzy extractors, registration centers, trusted hardware, or hybrid construction of classical and post-quantum mechanisms, which may increase system complexity and deployment cost. In addition, some recent studies have investigated network security from broader attack–defense perspectives, such as the combination of moving target defense and game theory, as well as game-theoretic strategy making for industrial defense scenarios. These studies highlight that secure communication systems increasingly operate in complex adversarial environments. Nevertheless, such defense-oriented approaches are complementary to, rather than replacements for, secure authentication and session key establishment protocols. Therefore, the main motivation of this work is to design a simpler two-party post-quantum authentication and key agreement protocol that can provide identity protection and secure session key establishment without introducing such auxiliary mechanisms. Compared with recent post-quantum authentication schemes based on Kyber or other lattice-based KEM primitives, the essential difference of the proposed protocol lies in its simplified two-party structure and its dual KEM-based session key establishment mechanism. In particular, unlike schemes that rely on fuzzy extractors, registration centers, trusted hardware, or hybrid constructions of classical and post-quantum mechanisms, the proposed design combines password verification, smart card-protected credentials, and Kyber-based encapsulation in a unified framework without introducing such auxiliary components. This difference leads to two concrete advantages. First, the protocol has lower structural complexity and is easier to deploy in conventional public network environments. Second, by avoiding fuzzy extractor-related processing and other auxiliary modules in the authentication phase, the proposed protocol achieves lower online computational cost while still preserving identity protection, mutual authentication, and post-quantum session key establishment.

## 3. Preliminaries

### 3.1. Module-LWE Assumption

Lattice-based cryptography relies on the hardness of Learning With Errors (LWEs)-type problems. The post-quantum KEM scheme derives its security from the Module-LWE (MLWE) assumption.

Let λ denote the security parameter. Let q=q(λ) be a prime modulus, and n=n(λ) and k=k(λ) be positive integers polynomial in λ. Define the polynomial ring $R_{q}=Z_{q}\left[X\right]/\left(X^{n}+1\right)$, and let $R_{q}^{k}$ denote the *k*-dimensional module over $R_{q}$. Let χ=χ(λ) be an efficiently samplable error distribution over $R_{q}$.

**Definition 1**  (Module-LWE Distribution). *Let $A\leftarrow R_{q}^{k\times k}$ be uniformly random. Let $s\leftarrow \chi^{k}$ and $e\leftarrow \chi^{k}$. The Module-LWE distribution $D_{MLWE}$ is defined as the distribution of pairs*

$$\left(A,b\right),\;\mathrm{where}\;b=As+e\in R_{q}^{k}.$$

**Definition 2**  (Uniform Distribution). *Let $A\leftarrow R_{q}^{k\times k}$ and $u\leftarrow R_{q}^{k}$ be sampled uniformly at random. The uniform distribution $D_{U}$ is defined as the distribution of pairs (A,u).*

**Definition 3**  (Decisional Module-LWE Problem). *Given a pair (A,v) sampled either from $D_{MLWE}$ or from $D_{U}$, the decisional Module-LWE problem is to determine from which distribution the pair was drawn.*

**Definition 4**  (MLWE Advantage). *For a probabilistic polynomial-time adversary A, its advantage in solving the decisional MLWE problem is defined as*

$$Adv_{A}^{MLWE}\left(\lambda \right)=\left|\mathrm{Pr}\left[A(A,As+e)=1\right]-\mathrm{Pr}\left[A(A,u)=1\right]\right|,$$

*where the probabilities are taken over the randomness of A, s, e, u, and the internal randomness of A.*

**Definition 5**  (MLWE Assumption). *For any probabilistic polynomial-time adversary A, the advantage $Adv_{A}^{MLWE}\left(\lambda \right)$ is negligible in the security parameter λ.*

The IND-CCA security of the adopted lattice-based KEM scheme is based on the hardness of the decisional Module-LWE problem.

### 3.2. Key Encapsulation Mechanism

A key encapsulation mechanism (KEM) is a public-key primitive that enables efficient and secure negotiation of a shared key over an insecure channel that is completely exposed to adversary surveillance.

A KEM scheme comprises three probabilistic polynomial time algorithms, defined as follows:

- $\left(pk,sk\right)\leftarrow KeyGen\left(1^{\lambda }\right)$: On input the security parameter λ, the key generation algorithm outputs a public key pk and a secret key sk.
- (c,K)←Encaps(pk): On input a public key pk, the encapsulation algorithm outputs a ciphertext *c* and a shared secret *K*.
- $K^{\prime }\leftarrow Decaps\left(sk,c\right)$: On input a secret key sk and a ciphertext *c*, the decapsulation algorithm outputs a shared secret $K^{\prime }$.

A KEM scheme is correct if, for all security parameters λ, for all key pairs (pk,sk) generated by $KeyGen(1^{\lambda })$, and for all (c,K) generated by Encaps(pk), it holds that

Decaps(sk,c)=K

except with negligible probability in λ.

A KEM scheme is said to be indistinguishable under adaptive chosen-ciphertext attacks (IND-CCA secure) if no probabilistic polynomial-time adversary can distinguish between a real encapsulated key and a uniformly random key, even when given access to a decapsulation oracle, except for the challenge ciphertext.

Formally, let $\left(pk,sk\right)\leftarrow KeyGen\left(1^{\lambda }\right)$. The adversary is given pk and access to a decapsulation oracle. After receiving a challenge pair $\left(c^{*},K_{b}\right)$, where $K_{b}$ is either the real shared secret or a random string, the adversary outputs a guess $b^{\prime }$. The advantage is defined as

$$Adv_{A}^{IND\text{-}CCA}\left(\lambda \right)=\left|\mathrm{Pr}\left[b^{\prime }=b\right]-\frac{1}{2}\right|.$$

If this advantage is negligible in λ, then KEM is IND-CCA secure.

In this study, we adopt the post-quantum Kyber key encapsulation mechanism (KEM) proposed by Bos et al. [9], whose security is based on the difficulty of the Module-LWE assumption. The Kyber KEM consists of three probabilistic polynomial time algorithms, namely, Kyber.KeyGen, Kyber.Encaps, and Kyber.Decaps.

## 4. Proposed Protocol

### 4.1. Initialization Phase

In this phase, the cloud server executes the system initialization algorithm to generate all the necessary public parameters and its long-term secret credentials. The detailed steps are described as follows:

1. The server *S* selects a security parameter λ that determines the security level of the proposed scheme and chooses a secure collision-resistant one-way hash function $h:{\left\{0,1\right\}}^{*}\to {\left\{0,1\right\}}^{l}$, where *l* denotes the fixed output length of the hash function.
2. *S* selects a long-term master secret key $s\in Z_{q}$, which will be used to bind the registered user identities during the authentication phase. Furthermore, *S* runs the post-quantum key encapsulation mechanism (KEM) key generation algorithm:
  $$\left(pk_{S_{j}},sk_{S_{j}}\right)\leftarrow Kyber.KeyGen\left(1^{\lambda }\right),$$
  where the following apply:
  - $pk_{S_{j}}$ denotes the public key of server *S*;
  - $sk_{S_{j}}$ denotes the corresponding secret key.
  $pk_{S_{j}}$ will be used by legitimate users to encapsulate a shared secret during the authentication phase, while $sk_{S_{j}}$ is kept confidential by the server.
3. *S* securely stores the long-term secrets $\left\{x,\;sk_{S_{j}}\right\}$ in its protected database, and publishes the following public parameters:
  $$\mathrm{PP}=\{\lambda ,\;pk_{S_{j}},\;Kyber.Encaps,\;Kyber.Decaps,\;h\left(\cdot \right)\}.$$

### 4.2. User Registration Phase

**Figure 2:**
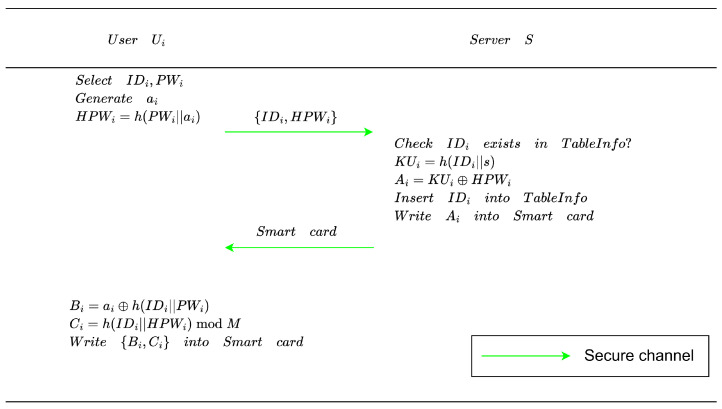
User registration phase.

User $U_{i}$ registers to the server *S* through a secure channel during this phase. The server maintains a registration database TableInfo and issues a smart card to the user. The process is illustrated in Figure 2, and its specific steps are described below:

1. $U_{i}$ selects an identity $ID_{i}$ and a password $PW_{i}$, and then generates a random nonce $a_{i}$. Next, $U_{i}$ computes $HPW_{i}=h(PW_{i}\left|\right|a_{i})$, and sends the registration request $\left\{ID_{i},\;HPW_{i}\right\}$ to *S* via a secure channel.
2. Upon receiving $\left\{ID_{i},HPW_{i}\right\}$, *S* checks whether $ID_{i}$ already exists in TableInfo. If $ID_{i}$ is not registered, *S* computes $KU_{i}=h(ID_{i}\left||s\right)$, and then derives $A_{i}=KU_{i}⊕HPW_{i}$. Thereafter, *S* inserts $ID_{i}$ into TableInfo, writes $A_{i}$ into a smart card SC, and sends SC to $U_{i}$ through the secure channel.
3. After receiving SC, $U_{i}$ computes $B_{i}=a_{i}⊕h(ID_{i}||PW_{i})$, and $C_{i}=h(ID_{i}||HPW_{i})\;\mathrm{mod}\;M$, where $2^{8}\leq M\leq 2^{10}$. Finally, $U_{i}$ stores $\left\{B_{i},C_{i}\right\}$ into SC to complete the registration.

After registration, the smart card finally stores the tuple $\left\{A_{i},B_{i},C_{i}\right\}$. Here, $A_{i}$ protects the server-related credential $K_{U_{i}}$ by combining it with the password-derived value $HPW_{i}$, while $B_{i}$ binds the local random nonce to the user’s identity and password, and $C_{i}$ is used for local password verification. Therefore, the data stored in SC do not expose either the password or $K_{U_{i}}$ in plaintext form, and the recovery of valid authentication parameters still depends on correct password input.

### 4.3. Login and Authentication Phase

**Figure 3:**
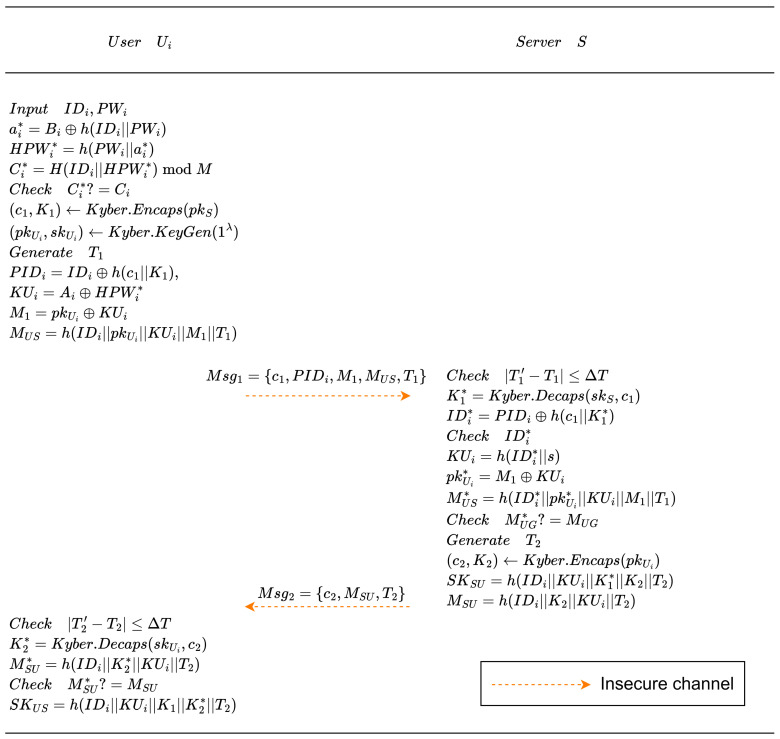
Authentication phase.

In this phase, the user $U_{i}$ and the server *S* mutually authenticate each other and derive a session key for securing subsequent communications. Figure 3 illustrates the computation and communication processes between the two parties during this stage. The specific procedure is as follows:

1. $U_{i}$ inserts SC into the terminal and provides $ID_{i}$ and $PW_{i}$. The terminal computes $a_{i}^{*}=B_{i}⊕h(ID_{i}||PW_{i})$, $HPW_{i}^{*}=h(PW_{i}\left|\right|a_{i}^{*})$, and $C_{i}^{*}=h(ID_{i}||HPW_{i}^{*})\;\mathrm{mod}\;M$. If the condition $C_{i}^{*}\neq C_{i}$ holds, the procedure is halted. Otherwise, $U_{i}$ evaluates $\left(c_{1},K_{1}\right)\leftarrow Kyber.Encaps\left(pk_{S}\right)$, where $pk_{S}$ denotes the public key of *S*. Subsequently, $\left(pk_{U_{i}},sk_{U_{i}}\right)\leftarrow Kyber.KeyGen\left(1^{\lambda }\right)$ is generated, where $pk_{U_{i}}$ and $sk_{U_{i}}$ represent the ephemeral public and secret keys of $U_{i}$, respectively. A fresh timestamp $T_{1}$ is produced, and the following values are derived: $PID_{i}=ID_{i}⊕h(c_{1}\left|\right|K_{1})$, $KU_{i}=A_{i}⊕HPW_{i}^{*}$, $M_{1}=pk_{U_{i}}⊕KU_{i}$, and $M_{US}=h(ID_{i}||pk_{U_{i}}||KU_{i}\left|\right|M_{1}\left|\right|T_{1})$. The authentication request $Msg_{1}=\left\{c_{1},PID_{i},M_{1},M_{US},T_{1}\right\}$ is then transmitted to *S* over the public channel.
2. Upon reception of $Msg_{1}$, the server verifies the freshness of $T_{1}$. If the timestamp falls outside the acceptable time window, the session is discarded. Otherwise, the server computes $K_{1}^{*}\leftarrow Kyber.Decaps\left(sk_{S},c_{1}\right)$, where $sk_{S}$ is the secret key of *S* and $c_{1}$ is the received ciphertext. Next, it recovers $ID_{i}^{*}=PID_{i}⊕h(c_{1}\left|\right|K_{1}^{*})$. After recovering $ID_{i}^{*}$, the server checks whether $ID_{i}^{*}$ exists in TableInfo; if not, the server will reject the request; otherwise, it calculates $KU_{i}^{*}=h(ID_{i}^{*}\left||s\right)$. The ephemeral public key is reconstructed as $pk_{U_{i}}^{*}=M_{1}⊕KU_{i}^{*}$. The server recomputes $M_{US}^{*}=h(ID_{i}^{*}||pk_{U_{i}}^{*}||KU_{i}^{*}\left|\right|M_{1}\left|\right|T_{1})$. A mismatch between $M_{US}^{*}$ and $M_{US}$ leads to termination; otherwise, user authentication succeeds. A new timestamp $T_{2}$ is generated, after which $\left(c_{2},K_{2}\right)\leftarrow Kyber.Encaps\left(pk_{U_{i}}^{*}\right)$ is obtained, where $pk_{U_{i}}^{*}$ denotes the reconstructed ephemeral public key of $U_{i}$. The server then derives $SK_{S}=h(ID_{i}^{*}||KU_{i}^{*}\left|\right|K_{1}^{*}\left|\right|K_{2}\left|\right|T_{2})$ and computes $M_{SU}=h(ID_{i}^{*}\left|\right|K_{2}||KU_{i}^{*}\left|\right|T_{2})$. Finally, the response message $Msg_{2}=\left\{c_{2},M_{SU},T_{2}\right\}$ is delivered to $U_{i}$.
3. After receiving $Msg_{2}$, $U_{i}$ examines the validity of $T_{2}$. An invalid timestamp results in immediate termination. Otherwise, $U_{i}$ performs $K_{2}^{*}\leftarrow Kyber.Decaps\left(sk_{U_{i}},c_{2}\right)$, where $sk_{U_{i}}$ denotes the ephemeral secret key and $c_{2}$ is the received ciphertext. Subsequently, $M_{SU}^{*}=h(ID_{i}\left|\right|K_{2}^{*}||KU_{i}\left|\right|T_{2})$ is computed. If $M_{SU}^{*}$ differs from $M_{SU}$, authentication fails; otherwise, the server is authenticated successfully and $U_{i}$ derives $SK_{U}=h(ID_{i}||KU_{i}\left|\right|K_{1}\left|\right|K_{2}^{*}\left|\right|T_{2})$.

Consequently, both parties establish a common session key $SK_{U}=SK_{S}=SK$. It is worth emphasizing that the smart card-derived parameter $K_{U_{i}}$ is tightly coupled with the Kyber-based key establishment process. In particular, $K_{U_{i}}$ is used to derive $M_{1}=pk_{U_{i}}⊕K_{U_{i}}$ and the authentication value $M_{US}$, which binds the user’s credential-related information to the ephemeral public key and the first encapsulation result $\left(c_{1},K_{1}\right)$. It is also included in the final session key derivation together with $K_{1}$ and $K_{2}$. Hence, the smart card is not an isolated storage component but an integral part of both authentication consistency and session key establishment.

### 4.4. Password Update Phase

$U_{i}$ is allowed to update the password locally without communicating with the server during this phase.

1. $U_{i}$ inserts SC into the terminal and provides $ID_{i}$, the current password $PW_{i}$, and a new password $PW_{i}^{new}$.
2. The terminal computes $a_{i}^{*}=B_{i}⊕h(ID_{i}||PW_{i})$, $HPW_{i}^{*}=h(PW_{i}\left|\right|a_{i}^{*})$, and $C_{i}^{*}=h(ID_{i}||HPW_{i}^{*})\;\mathrm{mod}\;M$. The stored value $C_{i}$ in the smart card is compared with $C_{i}^{*}$. If a discrepancy is detected, the password update process is aborted. Otherwise, the following values are derived sequentially: $KU_{i}=A_{i}⊕HPW_{i}^{*}$, $HPW_{i}^{new}=h(PW_{i}^{new}\left|\right|a_{i}^{*})$, $A_{i}^{new}=KU_{i}⊕HPW_{i}^{new}$, $B_{i}^{new}=a_{i}^{*}⊕h(ID_{i}||PW_{i}^{new})$, and $C_{i}^{new}=h(ID_{i}||HPW_{i}^{new})\;\mathrm{mod}\;M$. Finally, the smart card updates the stored parameters $\left\{A_{i},B_{i},C_{i}\right\}$ to $\left\{A_{i}^{new},B_{i}^{new},C_{i}^{new}\right\}$, respectively.

## 5. Security Analysis

### 5.1. Adversary Model

Our focus is on an adversary operating in probabilistic polynomial time (PPT) within the framework of the Dolev–Yao (DY) threat model. The model assumes that the adversary has full control over the public channel as follows:

- The adversary can arbitrarily intercept, modify, delay, replay, or even forge messages, and can simultaneously initiate multiple concurrent sessions to cross-utilize information streams from different protocol instances.
- All system parameters (including public keys in the public key infrastructure, protocol flow descriptions, and even algorithm specifications) are considered public resources that adversaries can freely obtain.
- When an adversary captures a user’s smart card, they may use side-channel technology to extract long-term keys or temporary state data stored on the card. This is a strong adversary model that does not rely on physical protection mechanisms to ensure security.
- When analyzing forward confidentiality, it is presumed that the adversary successfully obtains the private keys or system-level secret parameters (such as the master key) of all communicating entities after the completion of a protocol session.
- Although an adversary gains control of the network and may obtain the exposed data as described above, their capabilities are still strictly limited to computationally feasible levels. Specifically, except in extremely rare cases with a low probability of λ, an adversary cannot break the underlying lattice-based key exchange mechanism (KEM) in polynomial time. Furthermore, the secure hash function cannot be effectively reversed.

### 5.2. Formal Security Analysis

Within this section, we demonstrate that the proposed protocol achieves semantic security in the Real-or-Random (ROR) model under the random oracle assumption and the IND-CCA security of the foundational KEM.

#### 5.2.1. ROR Model

Let $U_{i}^{a}$ be the *a*-th instance of user $U_{i}$, and let $S^{b}$ be the *b*-th instance of the server *S*. Let A be a probabilistic polynomial-time (PPT) adversary that controls the public communication channel. The adversary can make the following oracle queries:

- $Execute(U_{i}^{a},S^{b})$: This query represents a passive attack and gives back the transcript from a normal run between $U_{i}^{a}$ and $S^{b}$.
- Send(Π,m): This query shows how active attacks work. The adversary sends message *m* to instance Π and gets the reply.
- Reveal(Π): If instance Π has accepted, this query returns the session key it established.
- Corrupt(S): Return the server’s long-term secret key $sk_{S}$.
- Test(Π): If Π is fresh and has accepted, a random bit c∈{0,1} is chosen. If c=1, the system returns the real session key; otherwise, it returns a random string that has the same length.

**Definition 6**  (Freshness). *An instance* Π *is fresh if the following apply:*

*1.*  Π *has accepted;*
*2.*  *Neither Reveal(Π) nor $Reveal(\Pi^{\prime })$ has been issued, where $\Pi^{\prime }$ is its partner;*
*3.*  *The server has not been corrupted before* Π *accepted.*

**Definition 7**  (Semantic Security). *Let c be the hidden bit used in the Test query and let $c^{\prime }$ be what the adversary outputs. We define the advantage of A against protocol P as*

$$Adv_{A}^{P}\left(t\right)=\left|2\mathrm{Pr}[c^{\prime }=c]-1\right|.$$

The protocol is semantically secure if $Adv_{A}^{P}\left(t\right)$ is negligible.

#### 5.2.2. Security Proof

**Theorem 1.**  *Assume that the KEM scheme is IND-CCA secure and the hash function h(·) works like a random oracle. Then the proposed protocol achieves semantic security in the ROR model.*

**Proof.**  We prove the theorem through a sequence of games. Let $Succ_{i}$ denote the event that the adversary A correctly guesses the hidden bit in Game $G_{i}$. The proof strategy is as follows. In Game $G_{1}$, the encapsulated secret $K_{1}$ is replaced with a uniformly random string, and the difference between $G_{1}$ and $G_{0}$ is bounded by the IND-CCA advantage of the underlying KEM. In Game $G_{2}$, the same argument is applied to the second encapsulated secret $K_{2}$. In Game $G_{3}$, we additionally exclude random oracle collisions and authentication forgeries without the corresponding hash queries. At the end of these transformations, the session key becomes computationally indistinguishable from a uniformly random string.

**Game $G_{0}$.** This game matches the real ROR experiment,

$$Adv_{A}^{P}\left(t\right)=2\mathrm{Pr}\left[Succ_{0}\right]-1.$$

**Game $G_{1}$.** In this game, for the fresh tested instance, the shared secret $K_{1}$ derived from $\left(c_{1},K_{1}\right)\leftarrow Kyber.Encaps\left(pk_{S}\right)$ is replaced with a uniformly random string. Since the tested instance is fresh, Corrupt(S) has not been issued before acceptance, and neither Reveal on the tested instance nor Reveal on its partner has been queried. Suppose that A can distinguish $G_{1}$ from $G_{0}$ with non-negligible advantage. Then one can build a PPT reduction algorithm $B_{1}$ against the IND-CCA security of the underlying KEM as follows: $B_{1}$ uses the IND-CCA challenge ciphertext and challenge key to simulate the pair $\left(c_{1},K_{1}\right)$ for the tested session, while answering all other oracle queries honestly using the public parameters and its own simulation. If the challenge key is real, the simulation is distributed as in $G_{0}$; if the challenge key is random, the simulation is distributed as in $G_{1}$. Therefore, any non-negligible advantage of A in distinguishing $G_{1}$ from $G_{0}$ yields a non-negligible IND-CCA advantage for $B_{1}$. Hence,

$$\left|\mathrm{Pr}\left[Succ_{1}\right]-\mathrm{Pr}\left[Succ_{0}\right]\right|\leq q_{exe}\cdot Adv_{KEM}^{IND\text{-}CCA}.$$

**Game $G_{2}$.** In this game, for the fresh tested instance, the shared secret $K_{2}$ derived from $\left(c_{2},K_{2}\right)\leftarrow Kyber.Encaps\left(pk_{U_{i}}\right)$ is replaced with a uniformly random string. Freshness guarantees that neither the tested instance nor its partner has been revealed, and that no corruption occurred before acceptance. Assume that A can distinguish $G_{2}$ from $G_{1}$ with non-negligible advantage. Then one can construct a PPT reduction algorithm $B_{2}$ against the IND-CCA security of the KEM by embedding the IND-CCA challenge pair into $\left(c_{2},K_{2}\right)$ for the tested session and simulating the remaining protocol execution consistently. If the challenge key is real, the view of A is distributed as in $G_{1}$; otherwise, it is distributed as in $G_{2}$. Therefore,

$$\left|\mathrm{Pr}\left[Succ_{2}\right]-\mathrm{Pr}\left[Succ_{1}\right]\right|\leq q_{exe}\cdot Adv_{KEM}^{IND\text{-}CCA}.$$

**Game $G_{3}$.** In this game, the experiment halts if a collision occurs in the random oracle. Let $q_{hash}$ denote the total number of hash queries and $l_{h}$ denote the output length of the hash function. By the birthday bound,

$$\left|\mathrm{Pr}\left[Succ_{3}\right]-\mathrm{Pr}\left[Succ_{2}\right]\right|\leq \frac{q_{hash}^{2}}{2^{l_{h}+1}}.$$

Furthermore, any successful forgery of authentication values without querying the corresponding random oracle input occurs with probability at most

$$\frac{q_{send}}{2^{l_{h}}}.$$

In Game $G_{3}$, both $K_{1}$ and $K_{2}$ are independent uniform values for the tested fresh session. Since the session key is computed as

$$SK=h(ID_{i}||KU_{i}||K_{1}||K_{2}||T_{2}),$$

and the hash function behaves as a random oracle, the session key is computationally indistinguishable from a uniformly random string. Thus,

$$\mathrm{Pr}\left[Succ_{3}\right]=\frac{1}{2}.$$

Combining the above inequalities, we obtain

$$Adv_{A}^{P}\left(t\right)\leq 2q_{exe}\cdot Adv_{KEM}^{IND\text{-}CCA}+\frac{q_{hash}^{2}}{2^{l_{h}}}+\frac{q_{send}}{2^{l_{h}}}.$$

Since the KEM scheme is IND-CCA secure and $l_{h}$ is sufficiently large, the advantage is negligible. □

### 5.3. Informal Security Analysis

In this subsection, we discuss several additional security and privacy properties of the proposed protocol at an informal level. In particular, anonymity and untraceability are argued based on the structure of the transmitted values and the assumed security of the underlying primitives, rather than through separate game-based privacy definitions.

#### 5.3.1. Anonymity

In the authentication phase, the user $U_{i}$’s real identity $ID_{i}$ is never sent as plain text. The user instead calculates a changing pseudonym $PID_{i}=ID_{i}⊕h(c_{1}\left|\right|K_{1})$. Here, $c_{1}$ is the KEM ciphertext and $K_{1}$ is the secret that was encapsulated. Because the KEM scheme is IND-CCA secure, $K_{1}$ looks random to any outside attacker. So $PID_{i}$ does not leak any details about the real identity $ID_{i}$. Therefore, the protocol provides a reasonable level of identity protection against outside observers at the informal analysis level.

#### 5.3.2. Untraceability

Each session uses fresh randomness from the KEM encapsulation $\left(c_{1},K_{1}\right)$, the ephemeral key pair $\left(pk_{U_{i}},sk_{U_{i}}\right)$, and the timestamp $T_{1}$. As a result, the pseudonym $PID_{i}$ and the transmitted messages change in every session, even for the same user. An adversary who observes multiple protocol runs cannot determine whether they come from the same user. Therefore, from the informal analysis perspective, the protocol provides resistance against straightforward user tracing across multiple sessions.

#### 5.3.3. Perfect Forward Secrecy

Suppose an adversary gets hold of the server’s long-term secret key $sk_{S}$ and the system secret parameter *s*. Moreover, assume that the adversary has recorded all earlier transmitted messages, including $c_{1}$, $c_{2}$, $PID_{i}$, $M_{1}$, $M_{US}$, and $M_{SU}$. Even with this strong assumption, the adversary still cannot recover previously established session keys. At the end of a session, the session key is calculated as $SK=h(ID_{i}||KU_{i}||K_{1}||K_{2}||T_{2})$. Here $K_{1}$ comes from the KEM output, that is, $\left(c_{1},K_{1}\right)\leftarrow Kyber.Encaps\left(pk_{S}\right)$ and $K_{2}$ are derived from $\left(c_{2},K_{2}\right)\leftarrow Kyber.Encaps\left(pk_{U_{i}}\right)$. After getting $sk_{S}$, the adversary can calculate $K_{1}=Kyber.Decaps\left(sk_{S},c_{1}\right)$ from the recorded ciphertext $c_{1}$. But recovering $K_{2}$ from $c_{2}$ needs the ephemeral private key $sk_{U_{i}}$ that corresponds to the temporary public key $pk_{U_{i}}$. In each session, the key pair $\left(pk_{U_{i}},sk_{U_{i}}\right)$ is newly created. The private key $sk_{U_{i}}$ is never stored or reused, so the adversary cannot get it. Without $sk_{U_{i}}$, trying to get $K_{2}$ from $c_{2}$ would break the IND-CCA security of the KEM scheme. Because SK depends on both $K_{1}$ and $K_{2}$, and $K_{2}$ remains computationally hidden, the adversary cannot recover previously established session keys even after it compromises the server’s long-term secret key. Therefore, the protocol provides forward secrecy.

#### 5.3.4. Mutual Authentication

When the server gets the login request, it first computes $K_{1}^{*}=Kyber.Decaps\left(sk_{S},c_{1}\right)$, $ID_{i}^{*}=PID_{i}⊕h(c_{1}\left|\right|K_{1}^{*})$, $KU_{i}^{*}=h(ID_{i}^{*}\left||s\right)$, and $pk_{U_{i}}^{*}=M_{1}⊕KU_{i}^{*}$. The server checks the authenticity of the request, verifying whether $M_{US}^{*}$ equals $h(ID_{i}^{*}||pk_{U_{i}}^{*}||KU_{i}^{*}||M_{1}||T_{1})$. To generate a valid $M_{US}$, an adversary must build a consistent tuple $\left(ID_{i},pk_{U_{i}},KU_{i},M_{1},T_{1}\right)$ such that the hash verification succeeds. However, computing the correct $KU_{i}$ requires knowledge of both the user’s identity and the secret value *s*, which is known only to the legitimate server. Moreover, constructing $M_{1}=pk_{U_{i}}⊕KU_{i}$ requires the correct $KU_{i}$, which in turn depends on valid credentials. An adversary needs the legitimate identity $ID_{i}$, the correct password $PW_{i}$ and the corresponding smart card data to derive the same parameters. Without them, they cannot generate a valid $M_{US}$. If someone changes or fakes the data, the verification will fail. So the server can confirm that the login request really comes from a legitimate user.

After receiving the login request, the legitimate server performs the following computations: $K_{1}^{*}=Kyber.Decaps\left(sk_{S},c_{1}\right)$, $ID_{i}^{*}=PID_{i}⊕h(c_{1}\left|\right|K_{1}^{*})$, and $KU_{i}^{*}=h(ID_{i}^{*}\left||s\right)$, where $sk_{S}$ is the server’s long-term private key and *s* is the server’s secret system parameter. Only a legitimate server that has both $sk_{S}$ and *s* can get the right $ID_{i}^{*}$ and calculate $KU_{i}^{*}$. Subsequently, the server generates $\left(c_{2},K_{2}\right)\leftarrow Kyber.Encaps\left(pk_{U_{i}}\right)$, and computes $M_{SU}=h(ID_{i}^{*}\left|\right|K_{2}||KU_{i}^{*}\left|\right|T_{2})$. The response message $Msg_{2}=\left\{c_{2},M_{SU},T_{2}\right\}$ is then sent to the user. Upon receiving $Msg_{2}$, the user computes $K_{2}^{*}=Kyber.Decaps\left(sk_{U_{i}},c_{2}\right)$, and verifies $M_{SU}^{*}=h(ID_{i}\left|\right|K_{2}^{*}||KU_{i}\left|\right|T_{2})$. To impersonate the server, an adversary must generate a valid pair $\left(c_{2},M_{SU}\right)$ that passes this verification. However, constructing a valid $M_{SU}$ requires the correct values of $KU_{i}^{*}$ and $K_{2}$. Deriving $KU_{i}^{*}$ requires knowledge of both the server secret parameter *s* and the correctly recovered identity $ID_{i}^{*}$, which itself depends on $K_{1}^{*}$. Recovering $K_{1}^{*}$ from $c_{1}$ requires the server’s private key $sk_{S}$. So, if an adversary does not have both $sk_{S}$ and *s*, it cannot compute $KU_{i}^{*}$ correctly. It also cannot create a consistent $M_{SU}$. Any fake response will fail the user’s verification. Consequently, the user can be sure the response message originates from the legitimate server.

#### 5.3.5. Session Key Agreement

The proposed protocol achieves authenticated session key agreement through contributions from both communicating parties. In the first message, the user computes $\left(c_{1},K_{1}\right)\leftarrow Kyber.Encaps\left(pk_{S}\right)$, where the shared secret $K_{1}$ can only be recovered by the legitimate server possessing the secret key $sk_{S}$. In the response phase, the server computes $\left(c_{2},K_{2}\right)\leftarrow Kyber.Encaps\left(pk_{U_{i}}\right)$, where the shared secret $K_{2}$ can only be recovered by the legitimate user through the ephemeral secret key $sk_{U_{i}}$. The final session key is derived as $SK=h(ID_{i}\|K_{U_{i}}\|K_{1}\|K_{2}\|T_{2})$. Therefore, the established session key depends on fresh secret values contributed by both sides rather than on the unilateral choice of a single participant. If either party fails to obtain the correct encapsulated secret, then the derived session keys at the two ends will be inconsistent, and the protocol execution will be rejected. Hence, the proposed scheme realizes authenticated session key agreement with joint entropy contribution from the user and the server.

#### 5.3.6. Post-Quantum Security

The post-quantum security of the proposed protocol mainly relies on the underlying Kyber key encapsulation mechanism, whose security is based on the hardness of the Module-LWE problem. In the authentication phase, the two secret values $K_{1}$ and $K_{2}$, which are essential for deriving the final session key, are established through Kyber.Encaps and Kyber.Decaps operations.

An adversary observing the public transcript may obtain the ciphertexts $c_{1}$ and $c_{2}$, but recovering the corresponding shared secrets from these ciphertexts requires breaking the confidentiality of the adopted lattice-based KEM. Under the assumed IND-CCA security of Kyber, such recovery is computationally infeasible even for adversaries with quantum capabilities. Since the session key is derived from $K_{1}$, $K_{2}$, and additional authentication parameters, an adversary cannot compute the established session key from the public transcript alone. Therefore, the proposed protocol provides post-quantum security for session key establishment.

#### 5.3.7. Resistance to Replay Attacks

The proposed protocol incorporates timestamps $T_{1}$ and $T_{2}$ into the exchanged authentication messages in order to guarantee message freshness. When the server receives $Msg_{1}=\left\{c_{1},PID_{i},M_{1},M_{US},T_{1}\right\}$, it first checks whether $T_{1}$ lies within the acceptable time interval. Therefore, if an adversary replays a previously intercepted login request after the valid time window has expired, the server will reject it immediately.

Similarly, after receiving $Msg_{2}=\left\{c_{2},M_{SU},T_{2}\right\}$, the user verifies the freshness of $T_{2}$. Thus, replaying an old response message cannot pass the freshness check on the user side. Moreover, the authentication values $M_{US}$ and $M_{SU}$ are bound to the corresponding timestamps and session-related parameters. Any attempt to modify a replayed message will lead to inconsistent verification results. Hence, the proposed protocol can effectively resist replay attacks in both communication directions.

#### 5.3.8. Resistance to Impersonation Attacks

To impersonate a legitimate user, an adversary must generate a valid login request $Msg_{1}=\left\{c_{1},PID_{i},M_{1},M_{US},T_{1}\right\}$ such that it can pass the server’s verification procedure. In particular, the adversary must construct mutually consistent values of $PID_{i}$, $M_{1}$, and $M_{US}$. However, $M_{1}=pk_{U_{i}}⊕K_{U_{i}}$ and $M_{US}=h(ID_{i}||pk_{U_{i}}\left|\right|K_{U_{i}}\left|\right|M_{1}\left|\right|T_{1})$, which both depend on the correct value of $K_{U_{i}}$. Deriving $K_{U_{i}}$ requires valid credential-related information associated with the legitimate user. Therefore, an external adversary cannot forge a valid login request without possessing the required secret information.

Similarly, to impersonate the server, an adversary must generate a response message $Msg_{2}=\left\{c_{2},M_{SU},T_{2}\right\}$ that can pass the user’s verification. For this purpose, the adversary must produce a valid encapsulated secret corresponding to the user’s ephemeral public key and compute $M_{SU}=h(ID_{i}\left|\right|K_{2}\left|\right|K_{U_{i}}\left|\right|T_{2})$. Without the ability to correctly process the user’s request and derive the required secret values, the adversary cannot generate a response consistent with the user’s verification equation. Hence, the proposed protocol resists both user impersonation and server impersonation attacks.

#### 5.3.9. Resistance to Man-in-the-Middle Attacks

In a man-in-the-middle attack, an adversary may intercept, modify, or replace the transmitted messages between the user and the server. However, the authentication request message is protected by the verification value $M_{US}=h(ID_{i}||pk_{U_{i}}\left|\right|K_{U_{i}}\left|\right|M_{1}\left|\right|T_{1})$. If the adversary modifies any component of $Msg_{1}$, such as $c_{1}$, $PID_{i}$, $M_{1}$, or $T_{1}$, then the server will derive inconsistent internal parameters, and the verification of $M_{US}$ will fail.

Likewise, the response message is protected by $M_{SU}=h(ID_{i}\left|\right|K_{2}\left|\right|K_{U_{i}}\left|\right|T_{2})$. If the adversary tampers with $c_{2}$, $M_{SU}$, or $T_{2}$, the user will obtain inconsistent values during decapsulation and verification, and the session will be rejected. Since both protocol messages are cryptographically bound to fresh secrets and authentication parameters, an adversary in the middle cannot alter the communication transcript while preserving successful verification at both ends. Therefore, the proposed protocol can effectively resist man-in-the-middle attacks.

#### 5.3.10. Resistance to Stolen Smart Card Attacks

In this attack scenario, we assume the adversary gets the user’s smart card and uses side-channel methods to extract all data stored on it. Even with full access to $A_{i}$, $B_{i}$, and $C_{i}$, the adversary still cannot directly recover the user’s password $PW_{i}$ or generate a valid authentication request. Specifically, the parameter $B_{i}$ is calculated as $a_{i}⊕h(ID_{i}||PW_{i})$, where $a_{i}$ comes from a random value. Without knowledge of $PW_{i}$, the adversary cannot recover $a_{i}$ correctly. Similarly, verifying a password guess requires computing $C_{i}^{*}=h(ID_{i}||HPW_{i}^{*})\;\mathrm{mod}\;M$, which depends on $HPW_{i}^{*}=h(PW_{i}\left|\right|a_{i}^{*})$. However, computing a consistent $a_{i}^{*}$ requires knowledge of the correct password. More importantly, the adversary must create a valid login request $Msg_{1}=\left\{c_{1},PID_{i},M_{1},M_{US},T_{1}\right\}$. The values of $PID_{i}$, $M_{1}$, and $M_{US}$ must agree with each other. Constructing a valid $M_{US}$ requires the correct value of $KU_{i}=A_{i}⊕HPW_{i}$, which depends on the correct $HPW_{i}$. Without knowing $PW_{i}$, the adversary cannot compute a valid $KU_{i}$, and therefore cannot generate a consistent $M_{1}$ or authentication value $M_{US}$. Any incorrect password guess will result in inconsistent hash computations, leading to verification failure at the server. So, even if someone fully breaks into the smart card and gets all the stored data, they still cannot make a valid login request unless they know the correct password. Therefore, the protocol is secure against stolen smart card attacks. The adversary model considered in this work allows smart card compromise via side channel attack. Under this assumption, the adversary may obtain the stored tuple $\left\{A_{i},B_{i},C_{i}\right\}$. However, such leakage does not immediately reveal the user’s password $PW_{i}$, the protected parameter $K_{U_{i}}$, or the session key. First, anonymity is still supported against passive outside observers because the transmitted pseudonym $PID_{i}=ID_{i}⊕h(c_{1}\left|\right|K_{1})$ depends on the fresh encapsulated secret $K_{1}$, which is not recoverable from the public transcript without breaking the underlying KEM. Second, forward secrecy is not invalidated by smart card leakage alone, because previously established session keys additionally depend on the fresh secret $K_{2}$ derived from the user’s ephemeral Kyber key pair; this value cannot be reconstructed without the corresponding ephemeral secret key $sk_{U_{i}}$. Third, session key security also does not follow from smart card leakage alone, since the final session key is derived from $K_{U_{i}}$, $K_{1}$, $K_{2}$, and fresh session data. Therefore, smart card compromise is a strong but still insufficient condition for breaking anonymity, forward secrecy, or session key security by itself.

#### 5.3.11. Resistance to Offline Password Guessing Attack

In this attack scenario, we assume an adversary obtains the user’s smart card and successfully extracts all stored parameters, including $A_{i}$, $B_{i}$, and $C_{i}$. Using this information, the adversary might try an offline password guessing attack. They would guess possible pairs $\left(ID_{i}^{\prime },PW_{i}^{\prime }\right)$ and then calculate $a_{i}^{\prime }=B_{i}⊕h(ID_{i}^{\prime }||PW_{i}^{\prime })$, followed by $HPW_{i}^{\prime }=h(PW_{i}^{\prime }\left|\right|a_{i}^{\prime })$, and finally $C_{i}^{*}=h(ID_{i}^{\prime }||HPW_{i}^{\prime })\;\mathrm{mod}\;M$. The adversary compares $C_{i}^{*}$ with the stored $C_{i}$ to check if the guessed pair $\left(ID_{i}^{\prime },PW_{i}^{\prime }\right)$ is correct. However, due to the modular operation in the computation of $C_{i}$, multiple distinct $\left(ID_{i},PW_{i}\right)$ pairs may produce the same value of $C_{i}$. In other words, the local verification condition $C_{i}^{*}=C_{i}$ is not uniquely binding to a single identity-password pair. So even if the guessed pair meets the local equality, the adversary still cannot be sure the guess is correct. To further verify whether the guess is valid, the adversary must interact with the server by launching an online login attempt. As long as the server has established a threshold for the number of consecutive failed login attempts, adversaries who repeatedly make incorrect guesses will quickly trigger the corresponding detection or account locking mechanisms. Hence, the proposed protocol can resist offline password guessing attacks.

#### 5.3.12. Resistance to Known Session-Specific Temporary Information Attack

In this attack scenario, it is assumed that the adversary obtains the temporary session related values generated during a completed protocol execution, including $c_{1}$, $K_{1}$, $c_{2}$, and $K_{2}$. Even with such leakage, the adversary still cannot directly compute the established session key.

The session key is derived as $SK=h(ID_{i}||K_{U_{i}}||K_{1}||K_{2}||T_{2})$. Therefore, besides the temporary values $K_{1}$ and $K_{2}$, the derivation of the session key still requires the correct protected parameter $K_{U_{i}}$. This value is associated with the legitimate user’s credentials and the server-side secret information, and it is not revealed by the temporary session transcript alone. Consequently, the exposure of session-specific temporary information does not enable the adversary to reconstruct the final session key. Hence, the proposed protocol is resistant to known session-specific temporary information attacks.

## 6. Comparison and Performance Evaluation

In this section, the proposed authentication protocol is compared with several representative related schemes, including Shamshad et al. [21], Luo et al. [8], Braeken et al. [18], and Wen et al. [22]. The comparison is conducted with respect to computational cost, communication overhead, and security properties. The selected comparison schemes are chosen because they are representative of closely related authentication designs from different but relevant perspectives. In particular, Shamshad et al. [21] and Luo et al. [8] represent lightweight classical authentication schemes with smart card or hardware-related protection mechanisms, while Braeken et al. [18] and Wen et al. [22] are more closely related post-quantum or hybrid authentication frameworks. Therefore, this comparison set allows us to evaluate the proposed protocol against both traditional lightweight baselines and recent quantum-resistant designs under the common criteria of computational cost, communication overhead, and security properties.

### 6.1. Computational Cost Analysis

To evaluate the computational efficiency of the proposed protocol, the execution time of the cryptographic operations involved in the authentication phase is analyzed. The running time of each cryptographic primitive used in the compared protocols is adopted from the experimental results reported in Wen et al. [22].

In [22], the execution time of the cryptographic operations was measured on a desktop computer running a 64-bit operating system, equipped with an Intel Core i5-12400F processor at 2.50 GHz and 18 GB RAM, with the implementation carried out using Python 3.9.1. Under this environment, the execution times for the various cryptographic operations are summarized in Table 1. Therefore, the present performance evaluation should be understood as an analytical comparison based on operation counts and reported unit costs, rather than as a direct implementation benchmark on a fixed hardware platform.

Based on the execution time listed in Table 1, the computational overhead of the authentication phase for each protocol can be derived. The comparison results are presented in Table 2.

The results in Table 2 can be explained by the types of cryptographic operations used in each scheme. In general, expensive operations such as elliptic-curve point multiplication and fuzzy-extractor-related processing contribute more to the online computational cost than hash evaluations and KEM-based operations. Since the proposed scheme avoids fuzzy extractors and other additional auxiliary modules in the authentication phase, its computational overhead is reduced accordingly. At the same time, for lattice-based schemes, the actual runtime in real deployments may vary significantly with the selected parameter set, implementation optimization level, and hardware environment. Therefore, the results in this section are intended to provide a comparative analytical indication rather than an exact platform-dependent measurement.

Although the compared schemes are not identical in every system assumption, they are sufficiently comparable in terms of authentication objective, security functionality, and online cost and thus provide a meaningful basis for evaluating the security efficiency trade-off of the proposed design.

The protocols proposed by Shamshad et al. [21] and Luo et al. [8] rely heavily on elliptic curve point multiplication operations, which incur relatively high computational overhead. Although Braeken et al. [18] adopted post-quantum cryptographic primitives, their computational overhead increased due to the introduction of additional symmetric encryption and PUF operations. Compared with the protocol proposed by Wen et al. [22], the protocol presented in this work eliminates fuzzy extractor operations and reduces the number of hash operations, thereby lowering computational overhead. This advantage comes not only from the use of lattice-based KEM primitives, but also from the simpler protocol structure adopted in the proposed design.

### 6.2. Communication Overhead Analysis

The metric for communication overhead is the total number of bits transmitted over the public channel during the authentication phase.

The dimensions of the various parameters used in the analysis are defined as follows: the hash value length is assumed to be 160 bits, the identity length is 40 bits, the nonce length is 40 bits, and the timestamp length is 32 bits. The length of the Kyber ciphertext *c* is assumed to be 1184 bits, while the length of the Kyber public key Pk is 1088 bits. The length of an elliptic curve point is assumed to be 256 bits, the length of a PUF challenge is 128 bits, and the output length of the symmetric encryption is assumed to be an integer multiple of 128 bits.

According to the authentication procedure, the proposed protocol exchanges two messages over the public channel:

$${Msg}_{1}=\left\{c_{1},PID_{i},M_{1},M_{US},T_{1}\right\}$$

$${Msg}_{2}=\left\{c_{2},M_{SU},T_{2}\right\}.$$

For $Msg_{1}$, the transmitted fields include $c_{1}$ (1184 bits), $PID_{i}$ (40 bits), $M_{1}$ (1088 bits), MUS (160 bits), and $T_{1}$ (32 bits). Therefore, $|{Msg}_{1}|\;=1184+40+1088+160+32=2504\;\mathrm{bits}$.

For $Msg_{2}$, the transmitted fields include $c_{2}$ (1184 bits), MSU (160 bits), and $T_{2}$ (32 bits). Therefore, $|{Msg}_{2}|\;=1184+160+32=1376\;\mathrm{bits}$.

Hence, the total communication overhead of the proposed protocol is 2504+1376=3880bits. Table 3 shows a comparison of the communication overhead of our proposed protocol with related protocols. The communication overhead of the proposed scheme is mainly affected by the ciphertext and public key-related parameters introduced by the lattice-based KEM. Although this overhead is higher than that of some classical lightweight schemes, it reflects the additional communication cost required to achieve post-quantum security.

### 6.3. Security Feature Comparison

Table 4 compares the security properties of the proposed protocol with several related schemes.

From Table 4, the proposed protocol satisfies all the evaluated security criteria. It ensures post-quantum security through the use of the Kyber lattice-based key encapsulation method, relying on the computational difficulty of the Module-LWE problem. Furthermore, the protocol guarantees user anonymity and untraceability, and effectively protects against numerous established threats such as replay attacks, impersonation attacks, man-in-the-middle attacks, stolen smart card attacks, offline password guessing attacks, and attacks targeting session-specific temporary information. These security results are consistent with the design of the proposed protocol. In particular, identity masking, dual encapsulated secrets, timestamp verification, and multi-factor protection jointly contribute to the achieved security properties. In this sense, the comparison is intended to show how the proposed protocol positions itself among representative related schemes, especially those pursuing similar authentication goals under stronger quantum-resistant security requirements.

## 7. Discussion

The proposed protocol employs the lattice-based Kyber key encapsulation mechanism, combined with password-based authentication and smart card protection mechanisms. This design endows the protocol with post-quantum security without sacrificing practical efficiency. The protocol implements several important security properties, including user anonymity, untraceability, two-way authentication, perfect forward confidentiality, and the ability to resist known attacks. By virtue of these characteristics, this scheme is highly suitable for secure authentication scenarios within modern network environments.

While keeping computational overhead at a relatively low level, the protocol also avoids the use of additional components such as fuzzy extractors or specialized hardware primitives. This design reduces implementation complexity and enhances the feasibility of practical deployment. Nevertheless, it is necessary to remain mindful of certain limitations inherent in the protocol. Ciphertexts generated by lattice-based cryptographic methods are typically longer than those produced by traditional cryptographic approaches, which may consequently increase communication overhead. Furthermore, because the protocol employs a timestamp mechanism to mitigate replay attacks, it imposes stringent requirements regarding the reliability of time synchronization. In light of this, appropriate time management strategies must be adopted in practical applications. In addition, although the manuscript informally discusses privacy-related properties such as anonymity and untraceability, a fully formal treatment under dedicated game based privacy definitions is left for future work.

Another limitation of the current work is that the protocol is developed for a basic two-party setting involving a single user and a single remote server. Although this model is useful for clearly studying the integration of password verification, smart card-protected credentials, and Kyber-based session key establishment, real cloud and IoT environments often involve multi-server, multi-domain, or federated authentication architectures. Extending the proposed design to such settings would require additional mechanisms for cross-server identity management, trust coordination, service migration, and key update procedures. These issues are not addressed in the present manuscript and should be regarded as important directions for future work.

Future research efforts could be further directed toward optimizing these post-quantum authentication protocols for resource-constrained devices and large-scale network environments, thereby rendering them more suitable for such settings. Concurrently, future work should therefore include an actual implementation of the proposed protocol and platform-specific benchmarking under different parameter sets, optimization strategies, and hardware environments, so as to complement the present analytical performance evaluation with real-world measurements.

## 8. Conclusions

This work presented a two-party post-quantum authentication and key agreement protocol based on the Kyber key encapsulation mechanism for secure network environments. By integrating password verification, smart card-protected credentials, and lattice-based KEM primitives, the proposed scheme enables mutual authentication and secure session key establishment over public channels.

Compared with several existing post-quantum authentication schemes, the proposed design avoids additional components such as fuzzy extractors, registration centers, and hybrid constructions of classical and post-quantum mechanisms, thereby reducing structural complexity. In particular, the protocol adopts a dual-KEM-based session key establishment mechanism, in which the final session key is derived from both a server-side encapsulated secret and an ephemeral encapsulated secret of the user. This design contributes to session key freshness, identity protection, and authenticated key agreement.

The security analysis indicates that the proposed protocol achieves semantic security in the ROR model under the random oracle assumption and the IND-CCA security of the underlying KEM while also providing anonymity, untraceability, replay resistance, impersonation resistance, man-in-the-middle resistance, and resistance to stolen smart card and offline password guessing attacks. The performance evaluation further shows that the proposed protocol achieves lower computational cost than several representative schemes because it avoids fuzzy extractor-related processing and other auxiliary modules in the authentication phase while maintaining acceptable communication overhead for post-quantum security.

Overall, the proposed scheme provides a practical and structurally simple approach to post-quantum authentication and key agreement in existing public network environments. At the same time, its communication cost remains influenced by the relatively large ciphertext and public key-related parameters of lattice-based KEM, which reflects a typical trade-off between post-quantum security and communication efficiency.

## Figures and Tables

**Table 1:** Execution time of cryptographic operations.

Symbol	Operation	Execution Time (ms)
$T_{h}$	Hash function operation	0.8
$T_{M}$	Elliptic curve point multiplication	51.5
$T_{SE}$	Symmetric encryption	14.3
$T_{SD}$	Symmetric decryption	14.3
$T_{KK}$	Kyber.Keygen operation	4.5
$T_{KE}$	Kyber.Encap operation	5.6
$T_{KD}$	Kyber.Decaps operation	7.0
$T_{FE}$	Fuzzy extractor operation	51.5
$T_{Bh}$	Biohashing operation	51.5
$T_{PUF}$	Physical unclonable function operation	0.5

**Table 2:** Computational overhead comparison (ms).

Protocol	User	Server
Shamshad et al. [21]	$7T_{h}+2T_{M}\approx 108.6$	$3T_{h}+2T_{M}+T_{SE}+T_{SD}\approx 134.0$
Luo et al. [8]	$6T_{h}+4T_{M}+T_{Bh}\approx 262.3$	$4T_{h}+5T_{M}\approx 260.7$
Braeken et al. [18]	$4T_{h}+2T_{M}+T_{KE}+T_{SE}+T_{SD}\approx 140.4$	$7T_{h}+2T_{M}+2T_{KD}+T_{SD}+T_{PUF}\approx 137.4$
Wen et al. [22]	$7T_{h}+T_{KK}+T_{KE}+T_{KD}+T_{FE}\approx 74.2$	$6T_{h}+T_{KE}+T_{KD}\approx 17.4$
Ours	$7T_{h}+T_{KK}+T_{KE}+T_{KD}\approx 22.7$	$5T_{h}+T_{KE}+T_{KD}\approx 16.6$

**Table 3:** Communication overhead comparison.

Protocol	Communication Overhead
Shamshad et al. [21]	1384 bits
Luo et al. [8]	1640 bits
Braeken et al. [18]	3352 bits
Wen et al. [22]	4192 bits
Ours	3880 bits

**Table 4:** Comparison of security features.

	Shamshad et al.	Luo et al.	Braeken et al.	Wen et al.	Ours
Anonymity	✓	✓	✓	✓	✓
Untraceability	✓	✓	✓	✓	✓
Mutual authentication	✓	×	✓	✓	✓
Perfect forward secrecy	✓	✓	×	✓	✓
Session key agreement	✓	✓	✓	✓	✓
Post-quantum security	×	×	✓	✓	✓
Resisting replay attacks	×	×	×	✓	✓
Resisting impersonation attacks	✓	×	✓	✓	✓
Resisting MITM attacks	✓	✓	✓	✓	✓
Resisting stolen smart card attacks	✓	✓	✓	✓	✓
Resisting offline password guessing attacks	✓	✓	✓	×	✓
Resisting known session-specific temporary information attacks	✓	✓	✓	✓	✓

## Data Availability

Data are contained within the article.
